# Expanded natural killer cells potentiate the antimyeloma activity of daratumumab, lenalidomide, and dexamethasone in a myeloma xenograft model

**DOI:** 10.1007/s00262-022-03322-1

**Published:** 2022-11-16

**Authors:** Jaya Lakshmi Thangaraj, Sung-Hoon Jung, Manh-Cuong Vo, Tan-Huy Chu, Minh-Trang Thi Phan, Kyung-Hwa Lee, Seo-Yeon Ahn, Mihee Kim, Ga-Young Song, Jae-Sook Ahn, Deok-Hwan Yang, Hyeoung-Joon Kim, Duck Cho, Je-Jung Lee

**Affiliations:** 1 Research Center for Cancer Immunotherapy, Chonnam National University Hwasun Hospital, Chonnam National University Medical School, Hwasun, Republic of Korea; 2 Department of Hematology-Oncology, Chonnam National University Hwasun Hospital, Chonnam National University Medical School, Hwasun, Republic of Korea; 3 Department of Molecular Medicine, Chonnam National University, Gwangju, Republic of Korea; 4 BioMedical Sciences Graduate Program, Chonnam National University, Gwangju, Republic of Korea; 5 Cell and Gene Therapy Institute (CGTI), Samsung Medical Center, Seoul, Republic of Korea; 6 Department of Pathology, Chonnam National University Medical School, Gwangju, Republic of Korea; 7 Department of Health Sciences and Technology, SAIHST, Sungkyunkwan University, Seoul, Republic of Korea; 8 Department of Laboratory Medicine and Genetics, Samsung Medical Center, Sungkyunkwan University School of Medicine, Seoul, Republic of Korea

**Keywords:** Natural killer cells, Multiple myeloma, Immunotherapy, Daratumumab

## Abstract

The development of new treatment agents in recent decades has significantly improved the survival of patients with multiple myeloma (MM). Nonetheless, MM remains an incurable disease; therefore, novel combination therapies are required. Natural killer (NK) cells are one of the safest immunotherapeutic options. In this study, we found that the anti-myeloma activity of expanded NK cells (eNKs) was improved by daratumumab, lenalidomide, and dexamethasone (DRd) in an MM xenograft mouse model. NK cells expanded from peripheral blood mononuclear cells collected from MM patients were highly cytotoxic against DRd pretreated tumor cells in vitro. To mimic the clinical protocol, a human MM xenograft model was developed using human RPMI8226-RFP-FLuc cells in NOD/SCID IL-2Rγ^null^ (NSG) mice. MM bearing mice were randomly divided into six groups: no treatment, eNK, Rd, Rd + eNKs, DRd, and DRd + eNKs. DRd significantly enhanced the cytotoxicity of eNKs by upregulating NK cell activation ligands and effector function. DRd in combination with eNKs significantly reduced the serum M-protein level and prolonged mouse survival. In addition, DRd significantly increased the persistence of eNK and homing to MM sites. These results show that the anti-myeloma activity of ex vivo-expanded and activated NK cells is augmented by the immunomodulatory effect of DRd in MM-bearing mice, suggesting the therapeutic potential of this combination for MM patients.

**Supplementary Information:**

The online version contains supplementary material available at 10.1007/s00262-022-03322-1.

## Introduction

The development of monoclonal antibodies has changed the treatment paradigm of multiple myeloma (MM). In the phase 3, POLLUX and CASTOR trials, daratumumab (Dara), lenalidomide, and dexamethasone (DRd) or Dara, bortezomib, and dexamethasone (DVd) significantly prolonged progression-free survival, compared with lenalidomide/bortezomib and dexamethasone (Rd/Vd), in relapsed refractory MM (RRMM) [1–3]. Because the results were superior to the results of other triplet regimens such as carfilzomib, lenalidomide, and dexamethasone (KRd) or ixazomib, lenalidomide, and dexamethasone (IRd) in RRMM, DRd has been recommended for the first relapse of MM not refractory to lenalidomide [1, 4, 5]. However, most patients eventually relapse despite these combination therapies and require subsequent therapy. In addition, MM develops at older ages, when patients are frail; thus, a combination therapy with enhanced efficacy and low toxicity is needed [6–9].

Natural killer (NK) cells are innate immune cells that mediate anti-tumor responses without prior sensitization [10–12]. Several clinical trials have proven the safety and efficacy of NK cells in MM and other malignancies [13, 14]. Therefore, NK cells are a potential immunotherapy for RRMM; however, high expression of NK inhibitory molecules and tumor microenvironmental factors in MM patients suppress the activity of NK cells [15–17]. We established a genetically modified K562 expressing OX40 ligand and membrane-bound (mb) interleukin (IL)-18 and IL-21 (K562-OX40L-mbIL-18/-21), capable of inducing robust expansion of NKs from MM patients with enhanced cytotoxicity against MM cells [18]. In addition to eNKs, we evaluated the effect of DRd in combination in vitro and in vivo*.* Dara is a CD38-targeting monoclonal antibody used clinically against MM and RRMM. Dara activates NK cells by inducing degranulation and antibody-dependent cell-mediated cytotoxicity (ADCC). However, Dara spares some of CD38-expressing immune cells such as T cells and NK cells, leading to immune depletion [19–23]. Adoptive transfer of ex vivo-expanded NK cells (eNKs) could overcome this issue [21, 24, 25]. In our previous study, eNKs anti-tumor activity and the survival of MM xenograft mice were greatly improved by the combination of DVd [26].

Lenalidomide, an immunomodulatory drug, is the backbone of most treatment regimens for MM [1, 27]. Lenalidomide improves NK cell effector function and proliferation in vitro and in vivo; it is used in combination regimens to enhance immune effector cell function [28–30]. In a phase 3 study, DRd improved the progression-free survival of RRMM patients, compared with Rd [1, 4]. Therefore, we hypothesized that eNK–DRd combination would enhance eNK function and persistence, while sensitizing MM cells to NK cell-mediated lysis by inducing NK cell-activating ligands on the surfaces of MM cells.

In this study, we evaluated whether DRd enhances the efficacy of adoptively transferred NK cells in an MM xenograft model. This treatment approach significantly improved disease-free survival and overall survival in MM-bearing mice, while reducing the serum M-protein level. DRd augmented the anti-myeloma effect of eNKs by attracting tumor-infiltrating NK cells to myeloma sites through upregulation of NK-activating ligands. These findings show that adoptive immunotherapy using eNKs with DRd can boost eNK activity in MM.

## Materials and methods

### Ethics declaration

All experimental procedures were approved by the Institutional Review Board of Chonnam National University Hospital. Blood samples of patients with MM were collected, with informed consent. Animal experiments were performed in accordance with protocols approved by the Chonnam National University Animal Use and Care Committee.

### Cell lines, cytokines, and drugs

The tumor cell lines (K562, U266, RPMI8226, and Raji) used in this study were purchased from the American Type Culture Collection (Manassas, VA, USA). Our recently established K562-OX40L-mbIL-18/-IL-21 cells were used to expand and activate NK cells [18, 31]. The cell lines and NK cells were cultured in RPMI-1640 medium supplemented with 10% heat-inactivated fetal bovine serum (Gibco) and 1% penicillin/streptomycin at 37 °C in a humidified 5% CO_2_ incubator. NK cells were expanded in the presence of recombinant human IL-2 and IL-15 (PeproTech, Rocky Hill, NJ, USA).

### Surface and intracellular staining for flow cytometry

The phenotypic characteristics of the eNKs assessed by percentage of NK activating and inhibitory receptors on the surface of the eNK were analyzed by flow cytometry. In brief, eNKs (2 × 10^5^ cells) were stained with fluorochrome-conjugated antibodies [CD3, CD56, CD16, NKp30, NKp44, NKp46, NKG2D, and NKG2C (BD Biosciences, USA)] for 15–20 min and 20,000 events/sample were acquired using a BD FACSCalibur. Tumor cells were pretreated with Dara (10 μg/mL; Janssen Pharmaceuticals, Johnson & Johnson, NJ, USA), lenalidomide (1 µM; Celgene), and dexamethasone (50 nM; Daewon Pharmaceuticals, Seoul, South Korea) for 24 h and stained with phycoerythrin-conjugated MICA, MICB, ULBP1, ULBP2, and Fas antibodies. Intracellular staining was performed using a BD Cytofix/Cytoperm™ Kit (BD Biosciences) to assess the interferon (IFN)-*γ*, granzyme-B, perforin, TRAIL, and FasL levels in DRd-pretreated eNKs by flow cytometry. Flow cytometric data were analyzed using FlowJo software (FLowJo LLC, USA).

### Ex vivo NK cell activation and expansion

MM patients’ peripheral blood mononuclear cells (PBMCs) were isolated using Lymphoprep solution. To expand NK cells, PBMCs were co-cultured with K562-OX40L-mbIL-18/-21 feeder cells previously irradiated with 100 Gy in RPMI-1640 medium supplemented with 10% fetal bovine serum, 1% penicillin/streptomycin, and 4 mM L-glutamine with 10 IU/mL recombinant human IL-2 until day 7. From day 7, the IL-2 concentration was increased to 100 IU/mL and recombinant human IL-15 (10 IU/mL) was added to the medium. NK cells were replenished with fresh cytokine-containing medium, every 2–3 days. The expanded NK cells from day 14 were considered mature NK cells (purity > 90% by phenotype analysis) and used for in vitro and in vivo experiments.

### NK cell proliferation assay

To assess lenalidomide-induced NK cell proliferation, PBMCs from patients with MM were cultured in RPMI-1640 medium containing 10% fetal bovine serum, 1% penicillin/streptomycin, and 4 mM L-glutamine in the presence of 1 µM lenalidomide alone or with 50 U/mL IL-2; the culture medium was changed every 2 days with lenalidomide or with IL-2 for 3 weeks. Lenalidomide-treated cells were stained with anti-human CD3 and CD56 antibodies to assess NK cell purity. To evaluate lenalidomide-induced eNK proliferation, D14 harvested eNKs were labeled with carboxyfluorescein diacetate succinimidyl ester (CFSE, Life Technologies), and cultured with 1 µM lenalidomide alone or with 50 U/mL IL-2 in RPMI-1640 medium containing 10% fetal bovine serum, 1% penicillin/streptomycin, and 4 mM L-glutamine. eNK proliferation was assessed by flow cytometry.

### Cytotoxicity assay

eNK cytotoxicity was measured using a flow cytometry-based cytotoxicity assay. Tumor cells were stained with CFSE (Life Technologies, Carlsbad, CA) and were treated with Dara (10 μg/mL), lenalidomide (1 µM), and dexamethasone (50 nM) for 24 h and then co-cultured with eNKs at various effector-to-target (E:T) ratios at 37 °C for 4 h in a 5% CO_2_ incubator. After incubation, 1 μL of propidium iodide (Life Technologies) was added prior to FACS acquisition and analyzed using a BD FACSCalibur; 10,000 events/sample were collected. The proportion of dead cells among CFSE-positive cells was calculated by deducting the proportion of spontaneously dead cells.

### Degranulation assay

To examine degranulation potential of the eNKs, DRd-pretreated target cells (K562, U266, RPMI8226, and Raji) or primary MM cells were co-cultured with eNKs at 1:1 effector-to-target cell (E:T) ratio with phycoerythrin-conjugated CD107a antibodies in 96-well U-bottom plates. At 1 h after co-culture, Brefeldin A and monensin (BD Biosciences) were added, and the cells were incubated for 3 h at 37 °C in a 5% CO_2_ incubator [32]. After 4-h incubation, the cells were stained for CD3 and CD56 for 15 min and analyzed using a BD FACSCalibur; 10,000 events/sample were collected. Flow cytometric data were analyzed using FlowJo software.

## eNK–DRd combination in the MM xenograft model

NOD/SCID IL-2Rγ^null^ (NSG) mice purchased from the Jackson Laboratory (Bar Harbor, MA, USA) were used to create a tumor xenograft model. The human MM xenograft model was established by intravenously injecting RPMI8226-RFP-FLuc cells (5 × 10^6^ cells per mouse) into 9–12-week-old male and female NSG mice. RPMI8226-RFP-FLuc cells were intravenously injected into NSG mice (n = 15 per group). Ten days after tumor inoculation, MM-bearing mice were divided into the following six groups: no treatment (phosphate-buffered saline control), Rd, eNKs, Rd + eNKs, DRd, and DRd + eNKs. Dara (8 mg/kg/day) and dexamethasone (0.6 mg/kg/day) were injected on days 10, 17, 24, and 31 by intraperitoneal and intravenous injections, respectively. Lenalidomide (1 mg/kg/day) was administered orally from day 10 for 5 consecutive days for 4 weeks, with a 2-day interval in each week. One day after Dara and dexamethasone treatment, freshly harvested eNKs (2 × 10^7^ cells per mouse) were injected on days 11, 18, 25, and 32. MM progression was monitored by bioluminescence imaging (BLI) in both dorsal and ventral views; mice were intraperitoneally injected with D-luciferin (150 mg/kg/mouse; PerkinElmer, Waltham, MA) 10 min before imaging, and imaged using the Night Owl System (Berthold Technologies, Bad Wildbad, Germany) [33]. Serum M-protein levels were quantified by human lambda free light chain (Bethyl Laboratories, USA) assays [34]. In vivo persistence and tumor infiltration of eNKs and myeloma clearance at various MM sites were assessed by flow cytometry.

### Quantification of cytokine levels in MM-bearing mice

Human immune effector and regulatory cytokines in mouse serum samples were evaluated by enzyme-linked immunosorbent assay (ELISA). Human IFN-*γ*, tumor necrosis factor (TNF)-α, IL-6, IL-10 (BD OptEIA™), and human granzyme-B and perforin (Mabtech AB, Stockholm, Sweden) levels in serum were quantified using ELISA kits.

### Statistical analysis

All data were analyzed using Prism 5 software (GraphPad Software Inc., San Diego, CA, USA). Statistical significance was determined using Student’s *t*-test or one-way analysis of variance. *P-*values < 0.05 were considered indicative of statistical significance. Data are expressed as means ± standard deviations or standard errors of the mean.

## Results

### DRd induces NK cell-activating ligands on tumor cells and primary MM cells in vitro

**Fig. 1:**
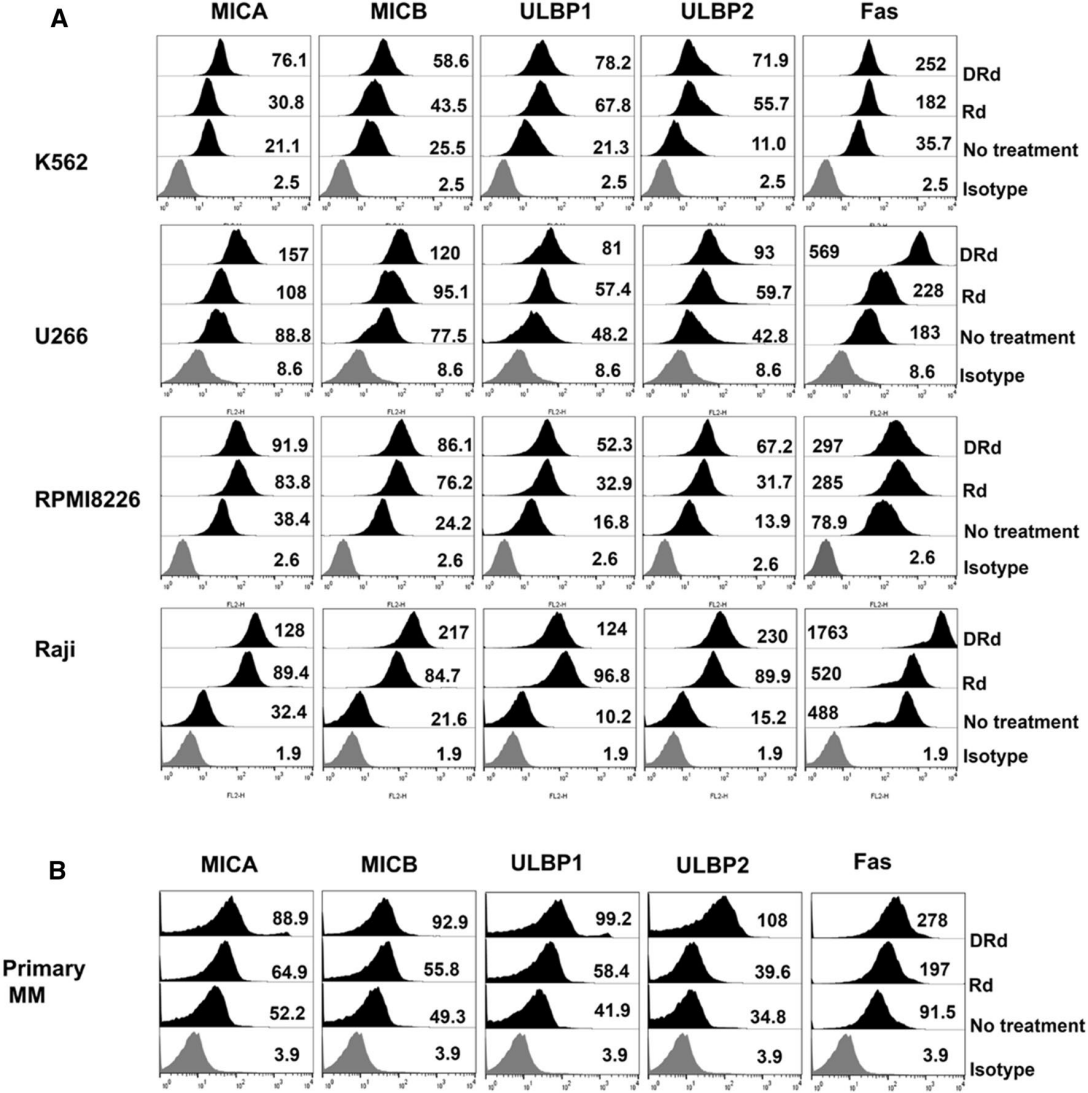
DRd increases NK-activating ligand expression on tumor cells. **A** and **B** Flow cytometry histograms showing surface expression (MFI value) patterns of MICA, MICB, ULBP1, ULBP2, and Fas on K562, U266, RPMI8226, Raji, and primary MM cells after treatment with Rd or DRd. DRd significantly increased the expression levels of MICA, MICB, ULBP1, ULBP2, and Fas receptor in both cell lines and primary MM cells. (See also Supplementary Fig. 2)

To investigate the effect of DRd on NK cell activation, we analyzed the expression patterns of NKG2D activating ligands and Fas receptor on the surfaces of K562, U266, RPMI8226, and Raji cells, as well as in primary MM cells. Tumor cells and primary MM cells treated with DRd for 24 h showed significantly increased expression of MICA, MICB, ULBP1, ULBP2, and Fas receptor compared to Rd or untreated tumor cells (all *p* < 0.0001, Fig. 1 and Supplementary Fig. 1). This indicates that DRd combination treatment will sensitizes tumor cells and increases NK-mediated tumor killing by upregulating NKG2D-activating ligands.

### DRd induced NK cell proliferation and enhances antitumor effects of eNKs in vitro

**Fig. 2:**
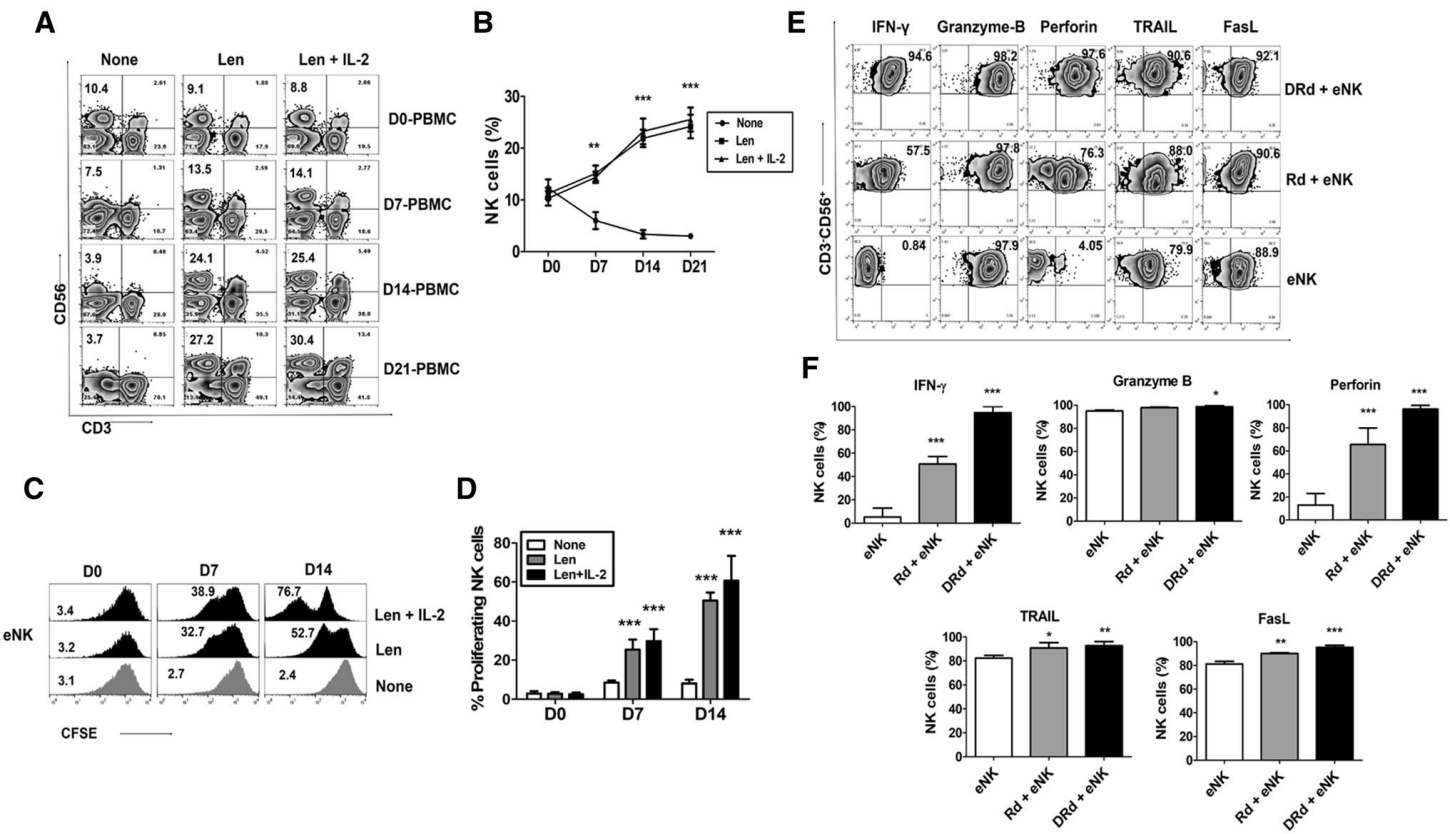
Effect of DRd on effector molecules expression in eNKs. **A** Representative flow cytometry plots of the expression of NK cell markers in peripheral blood mononuclear cells from patients with MM that had been treated with lenalidomide. **B** Mean ± standard deviation (SD) NK cell proportion in peripheral blood mononuclear cells from patients with MM (*n* = 5) after lenalidomide treatment. Lenalidomide significantly increased the NK cell proportion without cytokine stimulation. **C** Representative histogram showing the proliferation of CFSE-labeled eNKs treated with lenalidomide (1 µm) with or without IL-2. **D** Mean ± SD CFSE labeled eNK (*n* = 5) proliferation after lenalidomide treatment. Lenalidomide significantly induced eNK proliferation for 2 weeks with or without IL-2. **E** Representative FACS plot showing the expression of IFN-*γ*, granzyme-B, perforin, TRAIL, and FasL in eNKs. eNKs were harvested on day 14 and treated with DRd or Rd for 12 h. **F** Mean ± SD eNK (*n* = 5) expression of effector molecules after DRd treatment. DRd significantly increased the expression levels of IFN-γ and perforin in eNKs (mean ± standard deviation; *n* = 5). *p < 0.05, **p < 0.001, ***p < 0.0001

**Fig. 3:**
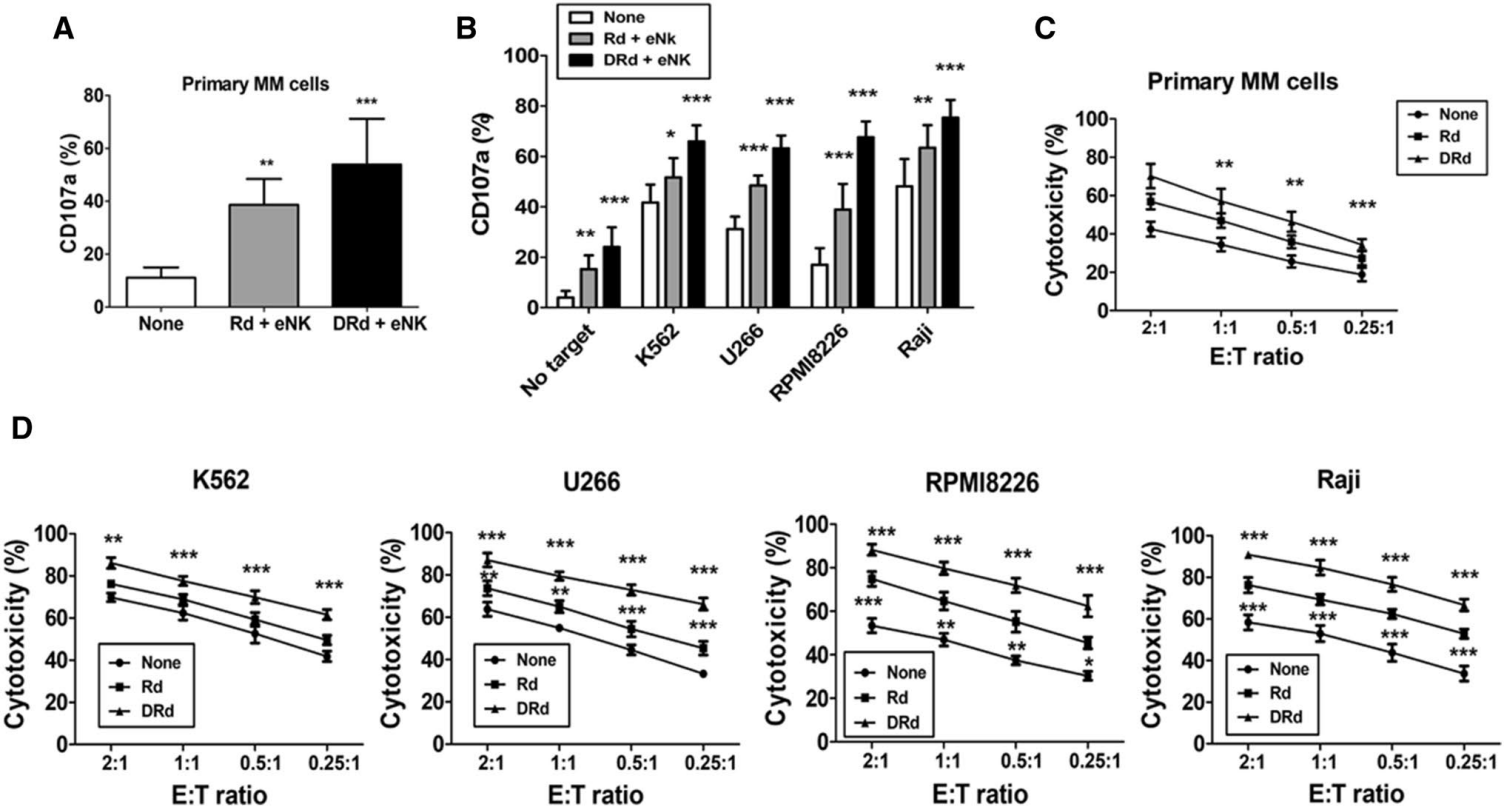
eNKs exert a strong cytotoxic effect on DRd-pretreated tumor cells and primary MM cells. (A and B) Proportion of CD107a-expressing eNKs (CD3^−^CD56.^+^) after co-culture with DRd-pretreated K562, U266, RPMI8226, Raji cells, or primary MM cells (E:T ratio of 1:1) for 4 h. Flow cytometry revealed a higher level of CD107a in eNKs that had been co-cultured with DRd-treated tumor cells. (C and D) eNK-mediated cytotoxicity in DRd-pretreated tumor cells (K562, U266, RPMI8226, and Raji) and primary MM cells measured using a standard 4-h flow cytometry-based cytotoxicity assay. eNKs showed potent antitumor activity against DRd-pretreated tumor cells at all E:T ratios. *p < 0.05, **p < 0.001, ***p < 0.0001

Lenalidomide has several immunomodulatory effects on various immune cells, including activation, proliferation, and direct tumor killing. We investigated the effect of lenalidomide on NK cell proliferation in MM patients. First, we evaluated lenalidomide-induced NK cell proliferation in MM patients’ PBMCs. Lenalidomide significantly increased the proportion of NK cells without cytokine stimulation in PBMCs of MM patients (Fig. 2A and B). Next, we assessed the lenalidomide-induced proliferation of eNK for 2 weeks with or without the addition of IL-2. eNK proliferation was significantly enhanced by lenalidomide (Fig. 2C and D). The impact of DRd on eNK effector functions was determined by evaluating the expression patterns of IFN-*γ*, granzyme-B, perforin, TRAIL, and FasL. DRd significantly increased the expression of IFN-*γ* and perforin in eNKs; however, the expression of granzyme-B, TRAIL, or FasL (Fig. 2E and F) is minimally affected with the treatment of either DRd or Rd combination. We evaluated the direct killing potential of DRd in tumor cells and primary MM cells. DRd alone was not enough to kill tumor cells or primary MM cells. Therefore, we assessed eNK-mediated cytotoxicity against DRd-pretreated tumor cells. Notably, DRd-pretreated tumor cells induced CD107a expression in eNKs (Fig. 3A and B). Furthermore, eNK cytotoxicity against DRd pretreated target cells was increased at all E:T ratios (Fig. 3C and D). These data suggest that DRd-pretreated tumor cells are sensitized to NK cells, which augments the antitumor effect of eNKs, thereby increasing cytotoxicity and presumably enhancing the anti-myeloma activity of eNKs.

### eNKs + DRd treatment exhibited improved anti-myeloma activity and prolonged survival in an RPMI8226-RFP-FLuc xenograft model

**Fig. 4:**
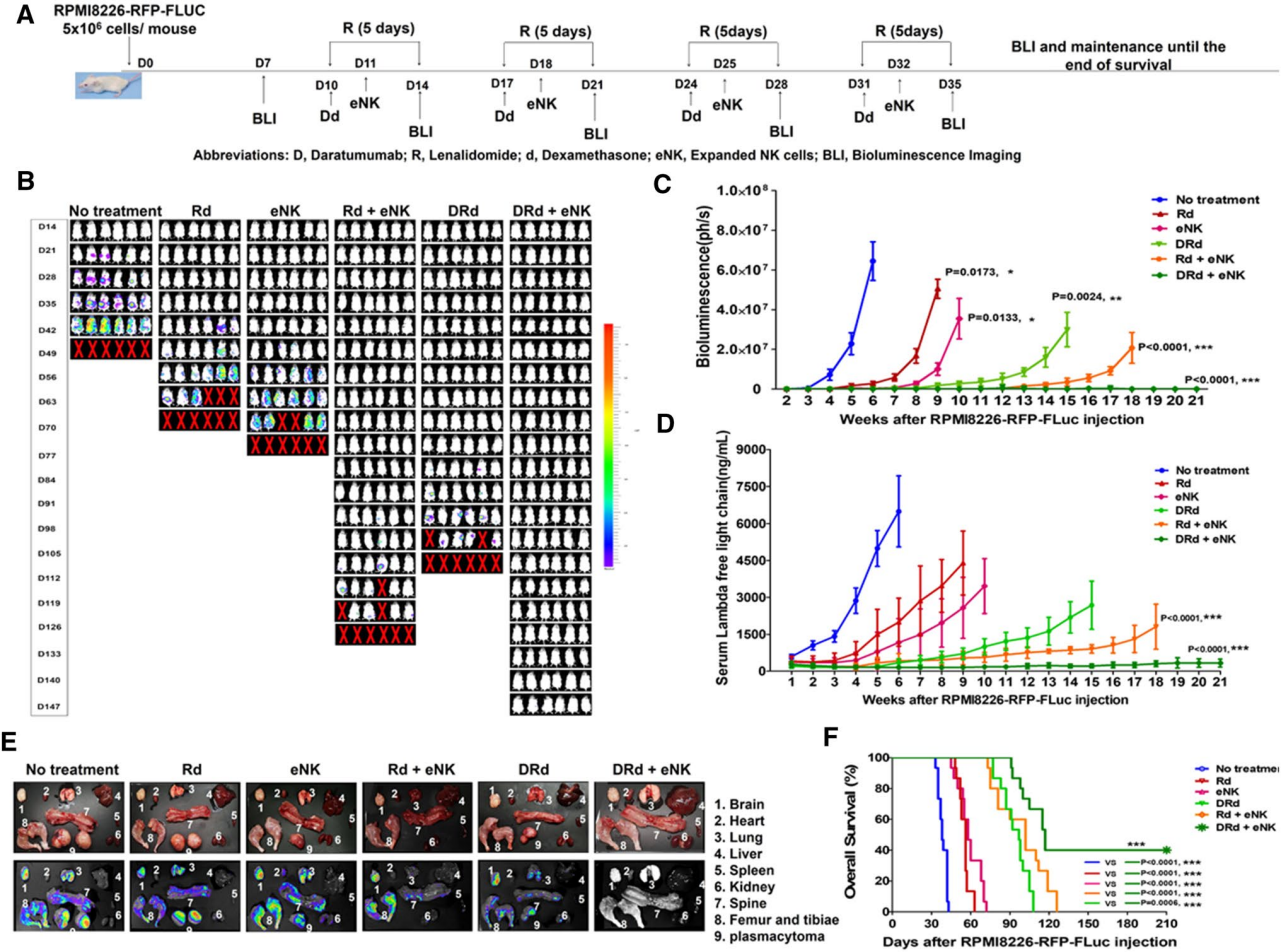
eNKs + DRd inhibited MM progression in RPMI8226-RFP-FLuc xenograft mice. **A** Treatment of RPMI8226-RFP-FLuc-bearing mice with eNKs + DRd. NSG mice were intravenously injected with 5 × 10.^6^ RPMI8226-RFP-FLuc cells, and tumor growth was monitored weekly by BLI. **B** Representative BLI of six mice (*n* = 15) from each group (dorsal view). **C** BLI showed that DRd + eNKs treatment provided the strongest antitumor effect at all time points. **D** Serum M-protein level determined by quantifying the level of human lambda free light chain in mouse peripheral blood. Mice treated with DRd + eNKs had the lowest serum M-protein levels. **E** Representative ex vivo BLI of mouse tissues. DRd + eNKs treatment did not result in a detectable bioluminescence signal. **F** Kaplan–Meier survival curves (*n* = 15 mice per group). Statistical significance was determined by log-rank test. Mice treated with DRd + eNKs exhibited the longest survival. *p < 0.05, **p < 0.01, ***p < 0.001

The above data indicated that DRd activates eNKs in vitro. Therefore, we investigated the importance of DRd combination with eNKs for the anti-myeloma effect in vivo, using the RPMI8226-RFP-FLuc xenograft model. Untreated MM-bearing mice showed rapid tumor growth, leading to death within 7 weeks. Mice in the Rd, eNKs, Rd + eNKs, and DRd treatment groups also showed significantly inhibited tumor growth but relapsed after treatment ended (Fig. 4A and B; Supplementary Fig. 3A). DRd + eNKs showed the strongest inhibition of MM progression throughout the survival period; six mice in the DRd + eNKs group were tumor free with no detectable bioluminescence (Fig. 4B and C) at all time points. Furthermore, none of the DRD + eNKs-treated mice had visible tumors or detectable serum M-protein (Fig. 4C and D, Supplementary Fig. 3B, Supplementary Table 1). Detectable and visible plasmacytomas were present in all other treatment groups, except the DRd + eNKs group (Fig. 4E and Supplementary Table 1). Furthermore, mice treated with DRd + eNKs exhibited the highest survival rate (Fig. 4F; ***, *p* < 0.001), and no significant body weight loss occurred in mice treated with Rd + eNKs or DRd + eNKs (Supplementary Fig. 3C). These results indicated that DRd + eNKs was capable of plasmacytoma and exert a long-term systemic anti-myeloma effect.

### eNK function, persistence, and homing increase with DRd treatment

**Fig. 5:**
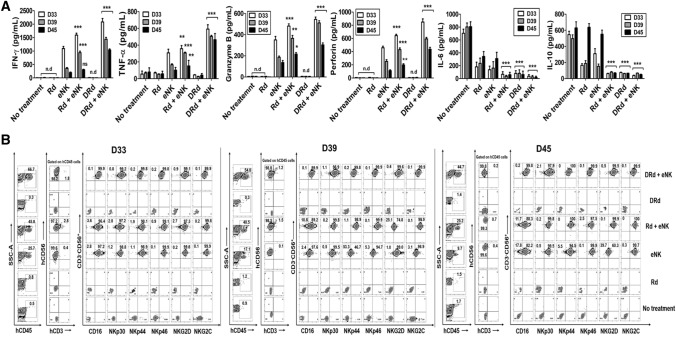
DRd enhanced the in vivo effector function and persistence of eNKs in RPMI8226-RFP-FLuc-bearing mice. **A** In vivo effector function of circulating eNKs, determined based on the levels of various immune effector and immunosuppressive cytokines at three time points from 1 day after final cell infusion (i.e., D33, D39, and D45). Mice treated with DRd + eNKs had the highest levels of IFN-*γ*, granzyme-B, perforin, and TNF-α, which decreased over time; they also had the lowest levels of IL-6 and IL-10. **B** Representative flow cytometry plots showing the in vivo persistence of circulating eNKs expressing NK activation receptors (CD16, NKp30, NKp44, NKp46, NKG2D, and NKG2C) among the hCD45 population with hCD3^−^CD56.^+^ expression at D33, D39, and D45. *p < 0.01, **p < 0.001, ***p < 0.0001

To investigate the in vivo effector function underlying the enhanced anti-myeloma effect of DRd combined with eNKs, we evaluated the serum levels of effector and immunosuppressive cytokines [35]. Mouse PB serum was collected at three time points (D33, D39, and D45), and the levels of human IFN-*γ*, granzyme-B, perforin, TNF-*α*, IL-6, and IL-10 (Fig. 5A) were measured. DRd + eNKs significantly increased the levels of effector cytokines IFN-*γ*, granzyme-B, perforin, and TNF-α at all time points; however, the effector cytokine levels gradually decreased over time. In addition, DRd + eNKs-treated mice showed the lowest levels of the immunosuppressive cytokines IL-6 and IL-10 at all time points. These data indicated that DRd + eNKs treatment not only improves in vivo effector function and also controls immunosuppressive cytokine secretion, thereby improving anti-myeloma activity in vivo. We also investigated the persistence of functional eNKs treated with DRd. The treatment group containing the eNK infusion showed significant eNK persistence in peripheral blood. The Rd + eNKs and DRd + eNKs groups had higher percentages of circulating eNKs than the eNKs alone group. Moreover, the phenotypic characteristics (CD16, NKp30, NKp44, NKp46, NKG2D, and NKG2C) of circulatory eNKs are remained unaffected in DRd + eNKs group compared to the eNKs alone or Rd + eNKs groups (Fig. 5B, Supplementary Fig. 4).

**Fig. 6:**
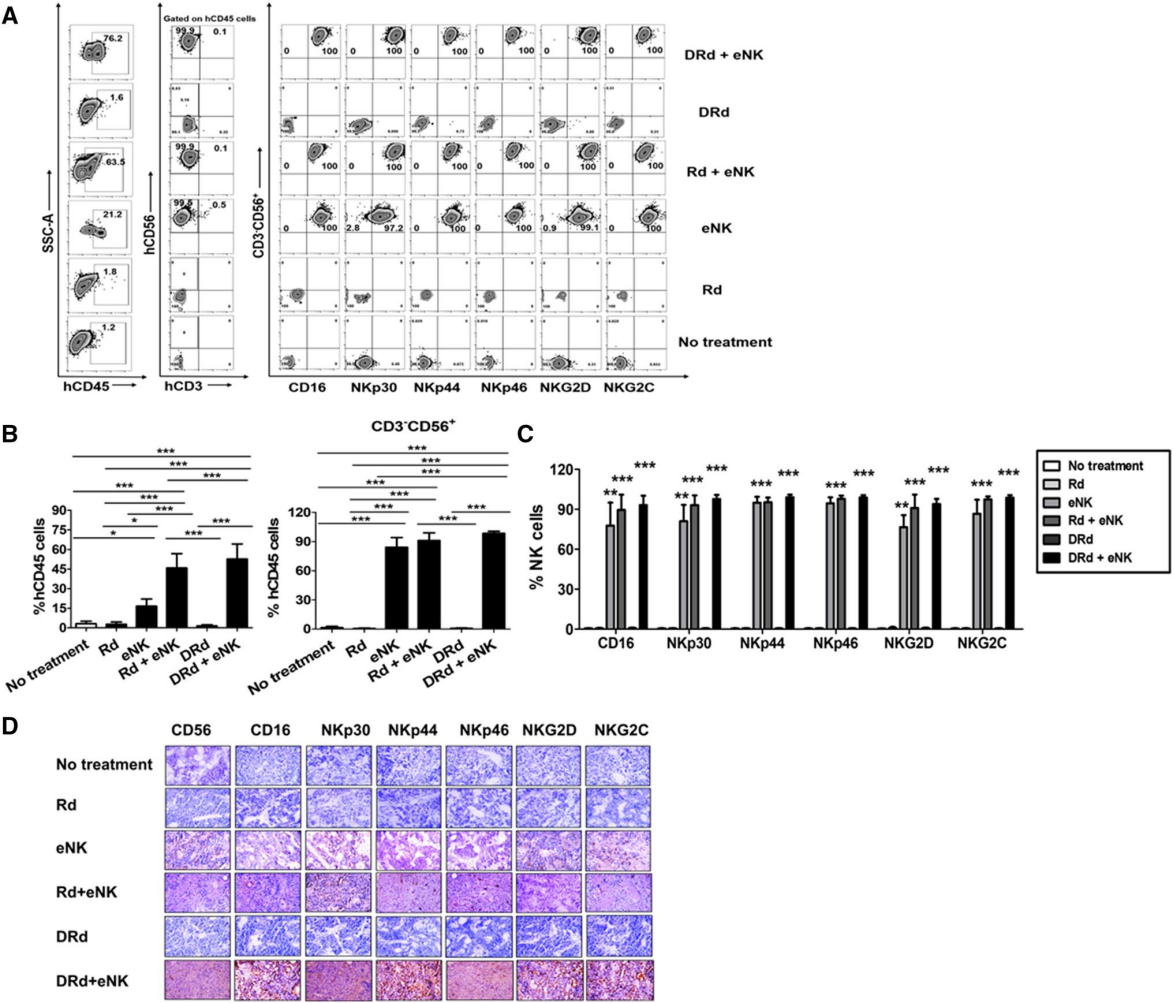
DRd enhanced eNK homing in vivo in RPMI8226-RFP-FLuc-bearing mice. **A** In vivo homing of eNKs in RPMI8226-RFP-FLuc-bearing mice (*n* = 10 per group) that were evaluated by flow cytometry. Mice were euthanized and BM samples were analyzed by flow cytometry for human NK cells (CD3^−^CD56^+^) and activation receptors (CD16, NKp30, NKp44, NKp46, NKG2D, and NKG2C) among the hCD45 population. (B, C) Quantification (mean ± SD) of in vivo eNK homing in the BM based on flow cytometry data. eNKs from mice treated with DRd + eNKs showed the greatest in vivo homing ability. *p < 0.01, **p < 0.001, ***p < 0.0001

Next, we investigated the in vivo homing and tumor-targeting abilities of eNKs in the RPMI8226-RFP-FLuc human MM xenograft model. After the experimental end point, tissue samples (bone marrow [BM], brain, heart, kidney, liver, lung, and spleen) were collected to evaluate the biodistribution of eNKs. DRd + eNKs-treated mice showed the greatest infiltration of eNKs in the BM (Fig. 6A), brain, heart, kidney, liver, lung, and spleen (Supplementary Fig. 5). We also evaluated the functional stability of eNKs by assessing NK cell purity and expression of activation receptors (CD16, NKp30, NKp44, NKp46, NKG2D, and NKG2C) in the BM (Fig. 6B and C) and other tissues (Supplementary Fig. 5). The eNKs infused in this xenograft model were highly stable in vivo which remained unaffected by the tumor microenvironment for an extended period.

**Fig. 7:**
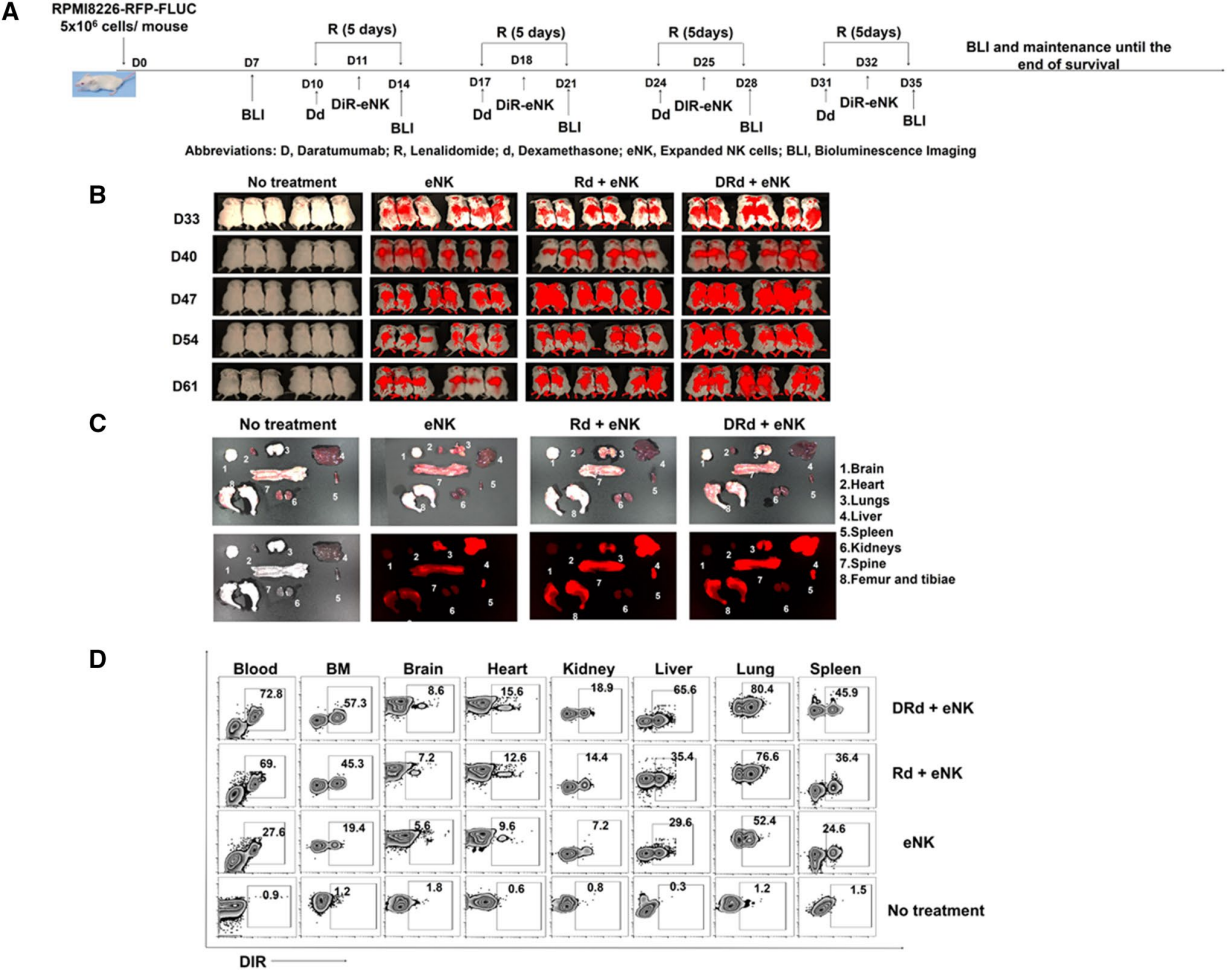
Trafficking of eNKs in DRd-treated RPMI8226-RFP-FLuc-bearing mice. (A) A separate set of RPMI8226-RFP-FLuc-bearing mice (*n* = 6 per group) were infused with DIR-labeled eNKs (2 × 10^7^ cells per mouse). (B) Treatment group mice were subjected to fluorescence imaging once weekly after the final eNK infusion for 5 weeks. **C** and **D** Mice were euthanized, and BM and other tissues were collected and subjected to fluorescence imaging and flow cytometry. eNK infiltration was greatest in the BM, liver, lung, and spleen

To evaluate eNK trafficking, mice in the eNKs, Rd + eNKs, and DRd + eNKs groups (*n* = 6 mice per group) received DIR (NIR dye)-labeled eNKs. DIR is a nontoxic near infrared dye that stains the cytoplasmic membrane of cells and is used to quantify the in vivo biodistribution of immune cells, in real time. eNK persistence and homing were assessed by real-time fluorescence imaging. Mice infused with DIR-labeled eNKs showed the greatest eNK homing to all tissues (Fig. 7A and B), and eNKs persisted until 5 weeks after eNK infusion. These findings were further confirmed by ex vivo fluorescence imaging and flow cytometry (Fig. 7C and D). eNK homing was more evident in the BM, liver, lung, and spleen compared with other tissues. Lenalidomide induced eNK persistence and homing, leading to NK proliferation and tumor infiltration. Therefore, the combination of Dara, lenalidomide, and dexamethasone increased the infiltration of eNKs into MM sites to a greater degree than Rd. Moreover, DRd combination treatment enhanced eNK-mediated tumor killing by suppressing the production of immunosuppressive cytokines in the MM microenvironment.

### eNKs + DRd treatment enhances MM clearance and NK-activating ligands in vivo in RPMI8226-RFP-FLuc xenograft model

**Fig. 8:**
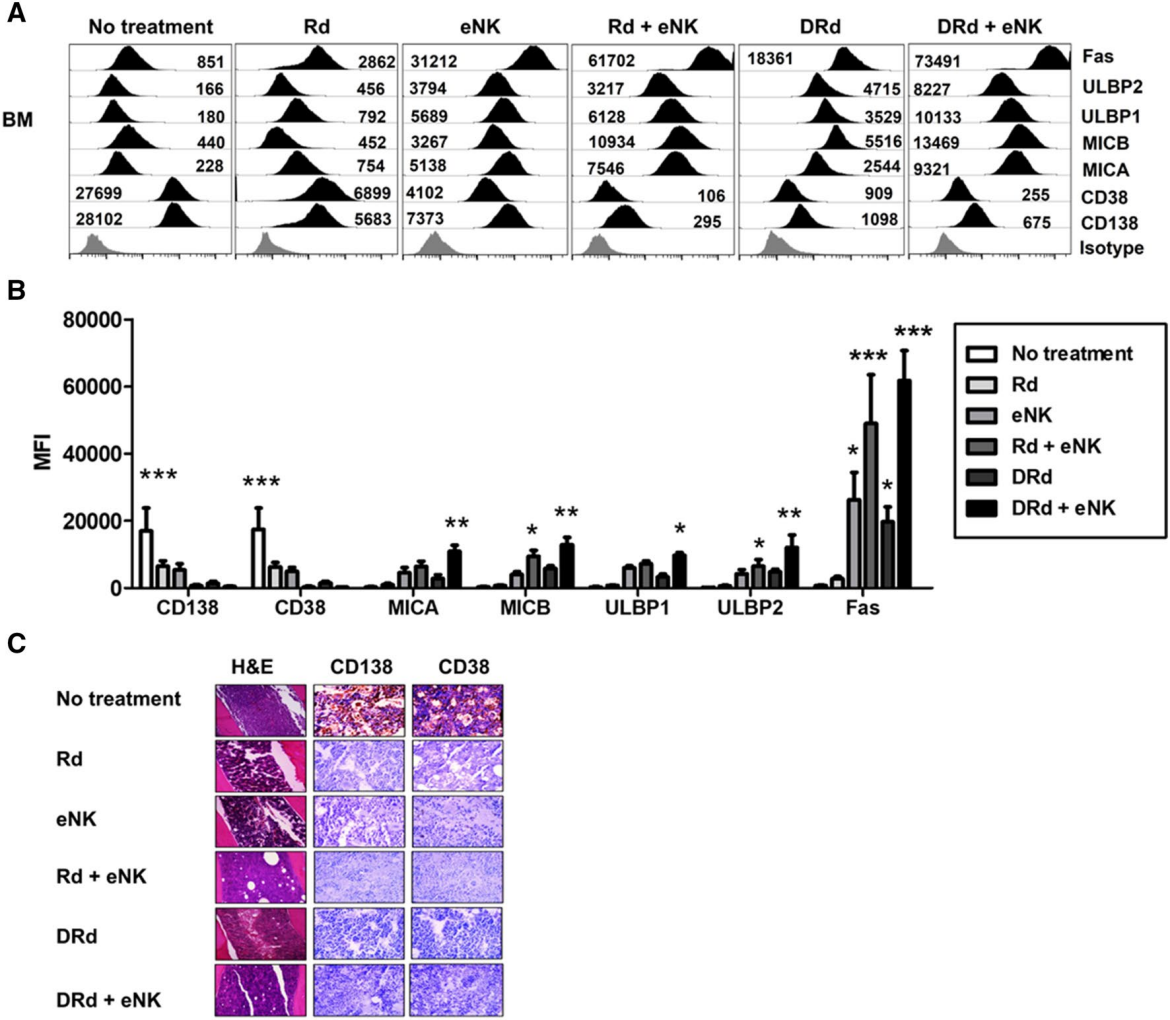
DRd + eNKs treatment enhances MM clearance and NK-activating ligand expression levels in RPMI8226-RFP-FLuc xenograft mice. **A** Residual RPMI8226-RFP-FLuc cells in the BM evaluated by flow cytometry. Representative histograms showing the expression levels (MFI value) of CD138, CD38, MICA, MICB, ULBP1, ULBP 2, and Fas. **B** Quantification (mean ± SD) of residual myeloma cells in the BM. Mice treated with Rd + eNKs and DRd + eNKs exhibited lower CD138 and CD38 expression levels in the BM, compared with the other groups; they also exhibited higher MICA, MICB, ULBP1, ULBP2, and Fas expression levels. *p < 0.01, **p < 0.001, ***p < 0.0001

The DRd + eNK-treated mice showed no visible signs of MM, no quantifiable serum M protein, and no detectable BLI signal. Therefore, we evaluated in vivo MM clearance and NK-activating ligand expression in all possible MM progression sites in the RPMI8226-RFP-FLuc MM xenograft model by flow cytometry. After the experimental end point, the mice were euthanized, and single-cell suspensions were generated from the BM, brain, heart, kidney, liver, lung, and spleen. The expression levels of MM markers (CD138 and CD38) and NK cell-activating ligands (MICA, MICB, ULBP1, ULBP2, and Fas) were evaluated by flow cytometry (Fig. 8A and B). Rd + eNKs and DRd with or without eNKs significantly cleared CD138 and CD38 cells in vivo*,* and DRd + eNKs significantly increased the expression levels of MICA, MICB, ULBP1, ULBP2, and Fas in all tissues (BM: Fig. 8A and B; other tissues, Supplementary Fig. 6).

## Discussion

NK cells are innate immune cells that kill tumor cells without prior sensitization, while avoiding the onset of graft-*versus*-host disease or other lethal adverse effects caused by CAR T cells [12–14]. Moreover, adoptive transfer of eNKs has several advantages over CAR T cells in terms of cancer immunotherapy [36–39]. However, the in vivo activity of NK cells can be suppressed by an immunosuppressive environment in the tumor; accordingly, combination treatments are needed to combat immunosuppression [14, 40]. We reported that Dara and bortezomib combination treatment significantly improved the anti-myeloma activity of eNKs [26]. In this study, we evaluated the ability of DRd to improve the in vivo effector function of adoptively transferred eNKs. DRd + eNKs treatment showed the greatest anti-myeloma effect on the in vivo eNK persistence and survival of RPMI8226-RFP-FLuc MM xenograft mice. DRd enhanced the anti-myeloma effect of eNKs in vivo by upregulating NK activating ligands, thereby enhancing eNK persistence, homing, and infiltration to myeloma sites.

Lenalidomide is an immunomodulatory drug that increases NK cell function, activation, and proliferation [28, 30, 41, 42]. Lenalidomide increases IFN-*γ*, granzyme B, and perforin production, as well as NK-mediated cytotoxicity and degranulation [29, 30, 43]. Moreover, lenalidomide in combination with monoclonal antibodies enhances the cytotoxicity of NK cells [1, 2, 24, 30]. Dara is a monoclonal antibody against CD38 that improves the function and killing potential of NK cells via ADCC. The anti-myeloma activity of eNKs was significantly increased by Dara in MM xenograft models [19, 21, 25, 44]. In this study, DRd + eNK upregulated NKG2D ligands in tumor cells and primary MM cells and induced NK cell proliferation and effector function, resulting in a superior anti-myeloma effect.

Cancer immunotherapy with the adoptive transfer of NK cells has several limitations such as a short lifespan, insufficient expansion, and poor in vivo persistence [39, 45, 46]. In general, a high dose of cytokines is administered to enhance the persistence of human NK cells in mouse models [32, 47–49]. However, the expression of membrane-bound IL-15 improved the survival and expansion of NK cells in vitro and in vivo without the administration of exogenous cytokines [50]. Additionally, IL-15-transduced cord blood-derived NK cells showed long-term antitumor activity and persistence in a mouse model of lymphoma [51]. In this study, we observed long-term circulatory maintenance of NK cells that had been expanded using genetically engineered K562-OX40L-mbIL-18/-21 feeder cells, in the absence of exogenous cytokines or CAR NK cells producing IL-15 [52]. Notably, DRd + eNKs-treated mice showed a significantly greater proportion of circulating eNKs than did mice treated with eNKs alone. Furthermore, DRd + eNKs-treated mice had the longest disease-free survival. Lenalidomide may significantly increase the NK cell proportion without cytokine stimulation. Importantly, graft-*versus*-host disease or cytokine release syndrome caused by the infusion of eNKs alone or in combination with DRd was not observed in our in vivo model. However, some mice were accidentally dead during the end point of study (90–100 days) without showing any signs of disease progression, and the reason for sudden death remains unclear.

CAR-T cell therapy targeting B-cell maturation antigen has demonstrated promising results for RRMM [53]. However, cytokine release syndrome and neurotoxicity mediated by proinflammatory cytokines are problematic and hamper the use of CAR-T cells in elderly patients with MM. CAR-NK cells could overcome these limitations. In recent phase 1 and 2 studies in lymphoma patients, CAR-NK cells did not cause significant cytokine release syndrome, neurotoxicity, or graft-*versus*-host disease; 73% of patients responded to CAR-NK cells within 30 days [37]. Although CAR-NK cells have shown promising anti-tumor activity in xenograft models and clinical studies, CAR gene transfer to NK cells is challenging [10, 32, 54]. The viral-vector transduction efficiency of CAR NKs is low, requires repeated transductions, and could alter NK function [10, 55, 56]. In addition, CAR-NK or CAR-T cell therapy is costly. Therefore, eNKs + DRd treatment may be more effective and tolerable in RRMM.

In conclusion, we investigated the potential of DRd to enhance the therapeutic efficacy of eNKs in vivo using an MM xenograft model. The anti-myeloma effect of eNKs was markedly enhanced by DRd combination treatment. These findings suggest that the eNKs plus DRd combination is a clinically feasible strategy for the treatment of MM.

## Supplementary Information

Below is the link to the electronic supplementary material.

Supplementary file1 (DOCX 4218 KB)

## Data Availability

The data generated in this study are available in the article and the supplementary data files.
