# Supplementary material for: Expanded natural killer cells potentiate the antimyeloma activity of daratumumab, lenalidomide, and dexamethasone in a myeloma xenograft model
Source: Cancer Immunol Immunother. 2022 Nov 16;72(5):1233–46. doi: 10.1007/s00262-022-03322-1 (PMC10110729; doi:10.1007/s00262-022-03322-1)
Supplement: Supplementary file 1 — Supplementary file1 (DOCX 4218 KB) [file 262_2022_3322_MOESM1_ESM.docx]

**Supplement table 1. The pathophysiological findings in RPMI8226-RFP-FLuc myeloma-bearing mice**

| Treatment group | Number of mice with paralysis  (total = 15 mice) | RPMI8226-RFP-FLuc BLI signal | | Serum M protein level  (human λ light chain) | Number of mice with plasmacytoma  (total = 15 mice) |
| --- | --- | --- | --- | --- | --- |
|  |  | Skeletal regions | Non-skeletal regions |  |  |
| No treatment | 15 | Very high | Low | Very high | 12 |
| eNK | 9 | High | Low | High | 7 |
| Rd | 10 | Very high | Low | Very high | 10 |
| Rd + eNK | 6 | Medium | Low | Low | 5 |
| DRd | 8 | Low | BDL | Medium | 8 |
| DRd + eNK | 5 | Very low | BDL | Very low | No |

Abbreviations: BLI, bioluminescence imaging; eNK, expanded natural killer cells; Dara, daratumumab; DRd, daratumumab, Lenalidomide, and dexamethasone; BDL, below the detection level.

**
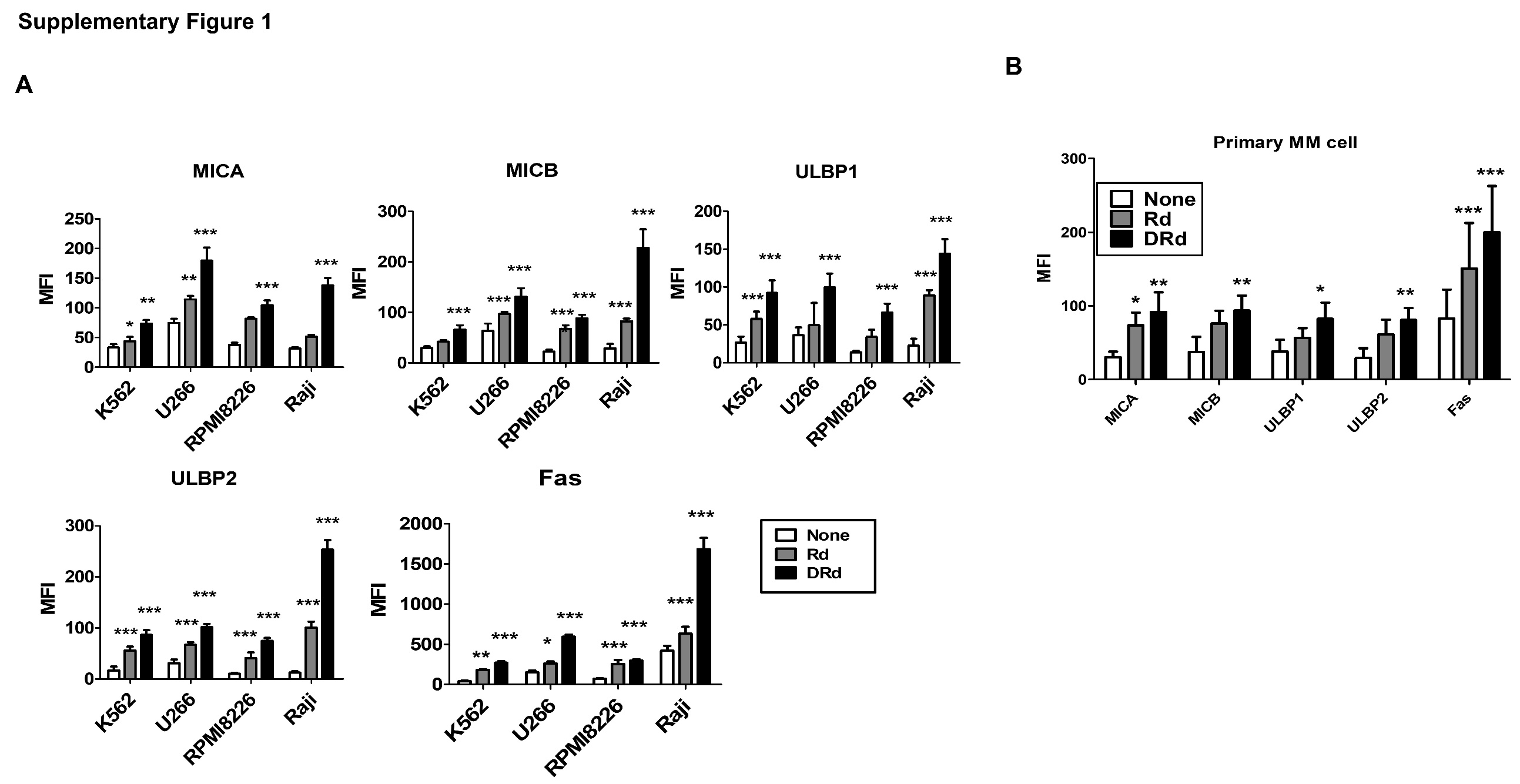
Supplementary Figure 1. DRd treatment downregulates NK inhibitory ligands and upregulates NK activating ligands in tumor cells.** (A, B) Mean ± SD quantification of surface expression (n = 5 independent experiments) of NKG2D ligands on tumor cells treated with DRd. Tumor cell lines (K562, U266, RPMI8226, and Raji cells) and primary myeloma cells were treated with 10 µM daratumumab, 1µM lenalidomide and 50 nm dexamethasone at indicated treatment combinations for 24 h, after that, the surface expression of NKG2D activating ligands and Fas receptor were analyzed by flowcytometry. DRd treatment significantly increased expression of MICA, MICB, ULBP1, ULBP2, and Fas receptor in both tumor cell lines and primary MM cells when compared to no treatment. *p < 0.05; **p < 0.001; ***p < 0.0001.


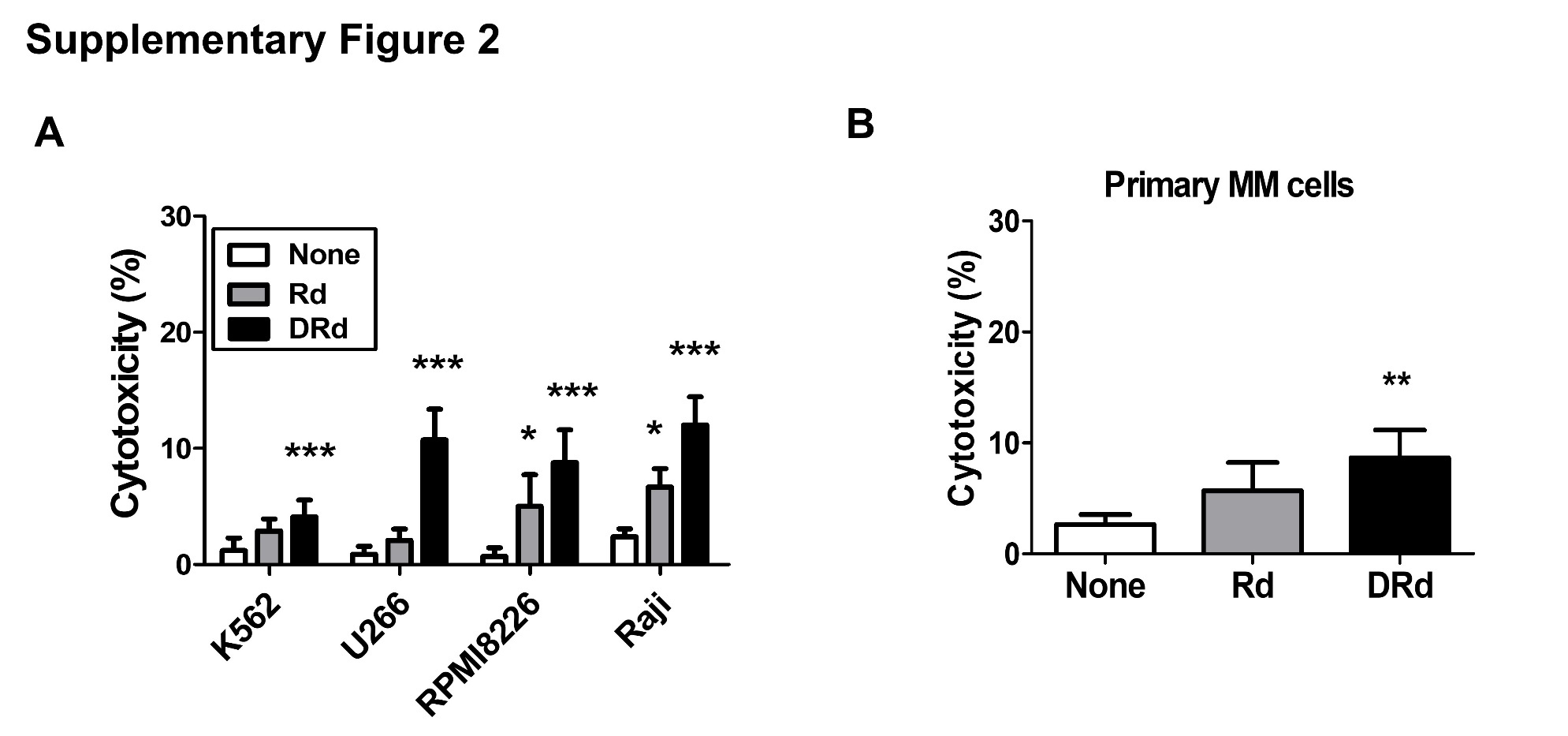


**Supplementary Figure 2.** **Cytotoxic effect of DRd treatment on tumor cell lines and primary MM cells. (A)** DRd-induced cytotoxicity in tumor cells were measured by flow cytometry-based cytotoxicity assay (mean ± SD; n = 5 independent experiments). Briefly, tumor cells (K562, U266, RPMI8226 and Raji) were labeled with CFSE as per manufactures protocol and CFSE-labelled tumor were treated with daratumumab (10 µg/mL), lenalidomide (1 µm) and dexamethasone (50 nm) for 24 h at indicated combinations. (B) Primary MM cells (n=7 donors) were also labeled with CFSE and treated with daratumumab, lenalidomide and dexamethasone for 24 h at indicated combinations. After that, the percentage cytotoxicity was measured by Flow cytometry. Lenalidomide alone not have much cytotoxicity effect in tumor cells (K562, U266, RPMI8226, and Raji) as well as primary MM cells. However, DRd combination treatment significantly induces the increased cytotoxicity in all tumor cells and primary MM cells. *p < 0.01, **p < 0.001, ***p < 0.0001.


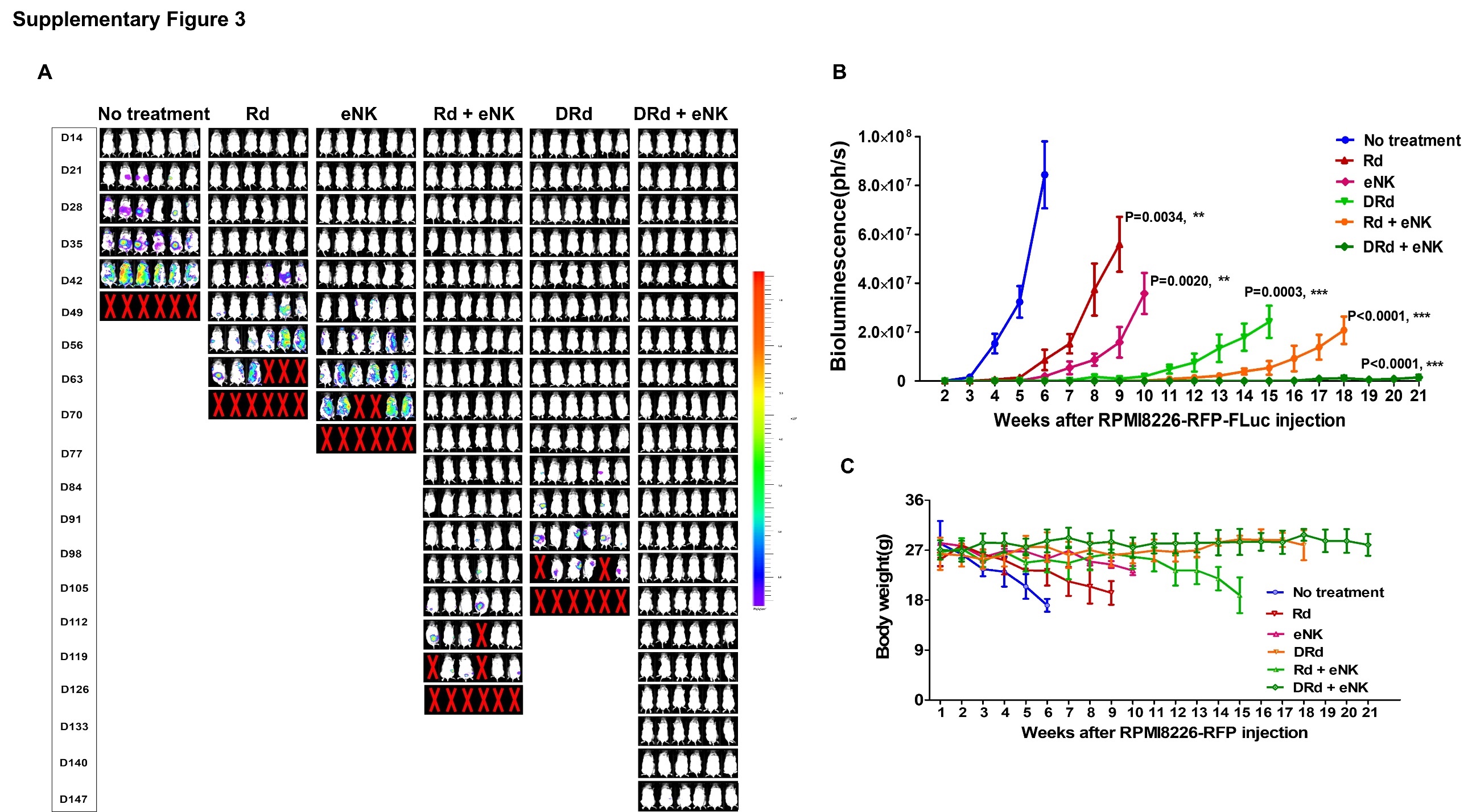


**Supplementary Figure 3.** **eNK combined with DRd exert superior anti-myeloma effects in RPMI8226-RFP-FLuc xenograft model.**  NSG mice were intravenously injected with 5 × 10^6^ RPMI8226-RFP-FLuc cells, and tumor growth was monitored weekly by bioluminescence imaging. Mice (n = 15 per group) were divided into six treatment groups as follows: no treatment (PBS control), Rd, eNK, Rd + eNK, DRd, and DRd + eNK, 10 days after tumor injection, mice were treated with DRd and eNK at indicated time points with appropriate drug combinations as described in methods. (A) Representative bioluminescence imaging of six mice from each group (Ventral view). (B) Graph showing the bioluminescence intensity in each group. Treatment with DRd + eNK provided the strongest antitumor effect at all time points. (B) Graphs showing the bioluminescence intensity measured weekly using the Night Owl System. Treatment with DRd + eNK provided the strongest anti-myeloma effect. (C) No significant body weight loss observed in mice treated with Rd + eNK and DRd + eNK. *p < 0.01, **p < 0.001, ***p < 0.0001.


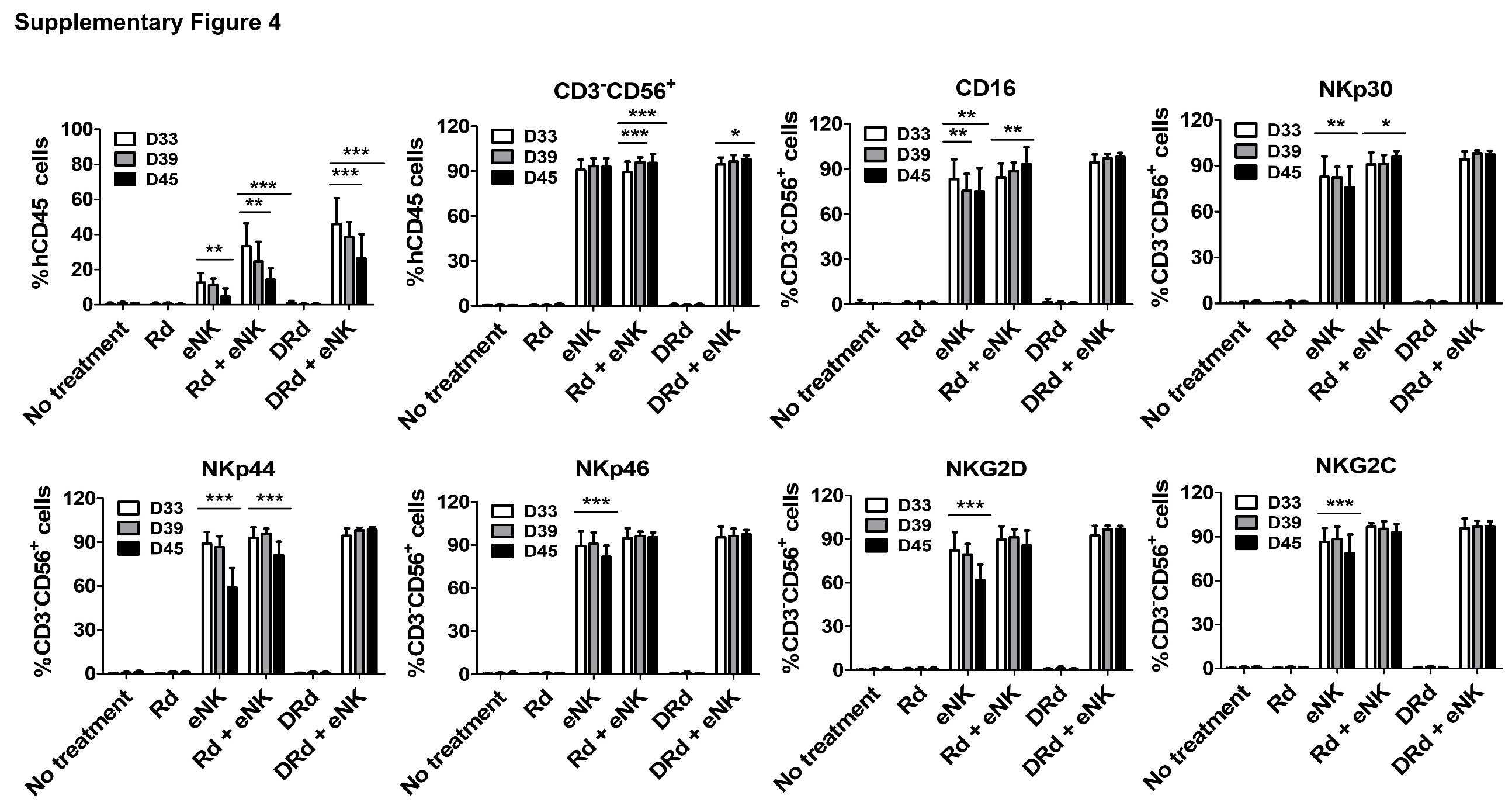


**Supplementary Figure 4.** **DRd pretreatment improves *in vivo* eNK persistence in RPMI8226-RFP-FLuc xenograft model.** *In vivo* persistence of circulating eNK in the RPMI8226-RFP-FLuc-bearing mice (n = 10 mice per group) were evaluated by flow cytometry. The graph represents the Mean ±SD quantification of *in vivo* persistence of circulating eNK and its activation receptors (CD16, NKp30, NKp44, NKp46, NKG2D, and NKG2C) based on flow cytometry data. The percentage of human NK cells (CD3^-^CD56^+^ cells) analyzed within the hCD45 population and the percentage of NK activation receptors (CD16, NKp30, NKp44, NKp46, NKG2D, and NKG2C) expression assessed within the CD3^-^CD56^+^ cells population from the mice peripheral blood collected at various time points (D33, D39, and D45). All treatment group mice infused with eNK showed significantly detectable eNK persistence in mouse peripheral blood, and Rd + eNK and DRd + eNK treatment group mice showed very high percentage of circulating eNK cells when compared to other treatment groups. Importantly, DRd + eNK treated mice displayed a significantly higher percentage of NK activation receptors (CD16, NKp30, NKp44, NKp46, NKG2D, and NKG2C) in the circulatory eNK compared with mice treated with either eNK alone or with Rd + eNK *p < 0.01, **p < 0.001, ***p < 0.0001.


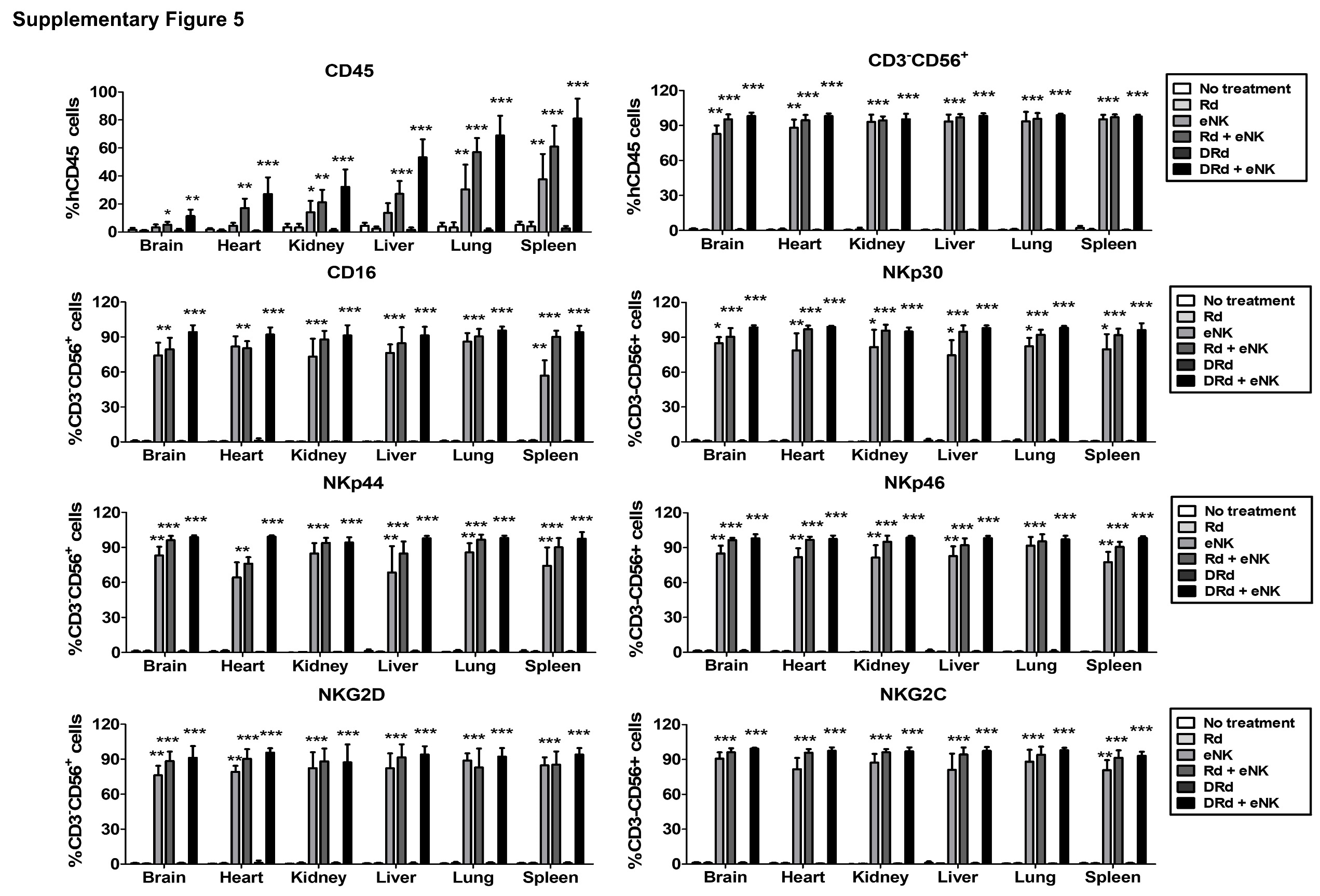


**Supplementary Figure 5.** *In vivo* homing of eNK in RPMI8226-RFP-FLuc-bearing mice (n = 10 mice per group) evaluated by flow cytometry. Mice were sacrificed at the experimental endpoint, brain, heart, kidney, liver, lung, and spleen samples were collected and analyzed by flow cytometry for human NK cells (CD3^-^CD56^+^) and activation receptor (CD16, NKpp30, NKp44, NKp46, NKG2D, and NKG2C) levels. Percentage of human NK cells were analyzed within the hCD45 population, from the human NK cells population the percentage of NK cells expressing NK activation receptors measured. The graph represents the quantification (mean ± SD) of *in vivo* eNK homing in the brain, heart, kidney, liver, lung, and spleen based on the flow cytometry data. DRd + eNK showed the highest *in vivo* homing to all tissues and highest expression of NK activating receptors than other treatment groups. DRd + eNK group showed the highest *in vivo* eNK homing and higher percentage of NK activating receptors in all tissues than other treatment groups. *p < 0.01, **p < 0.001, ***p < 0.0001.


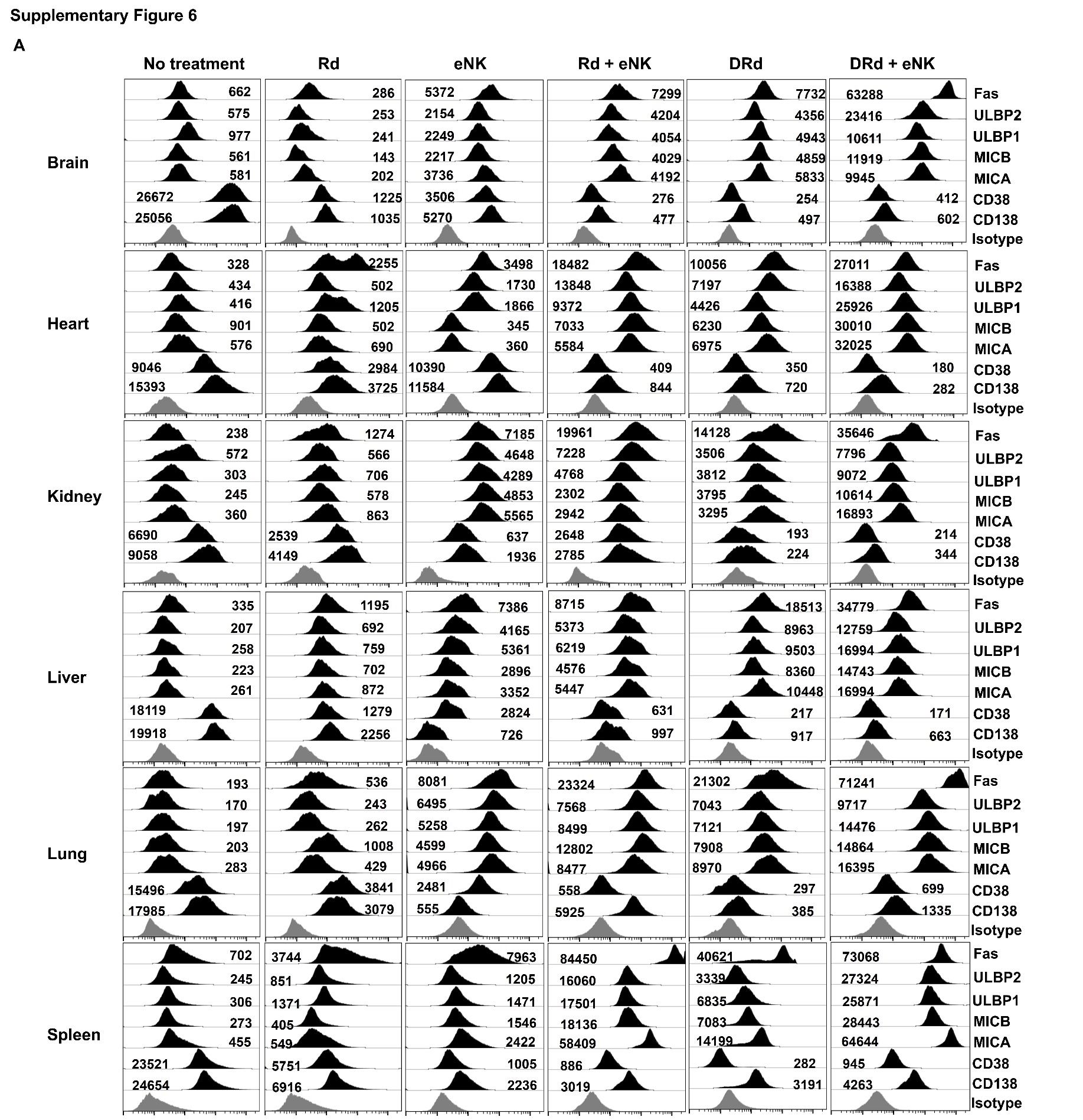


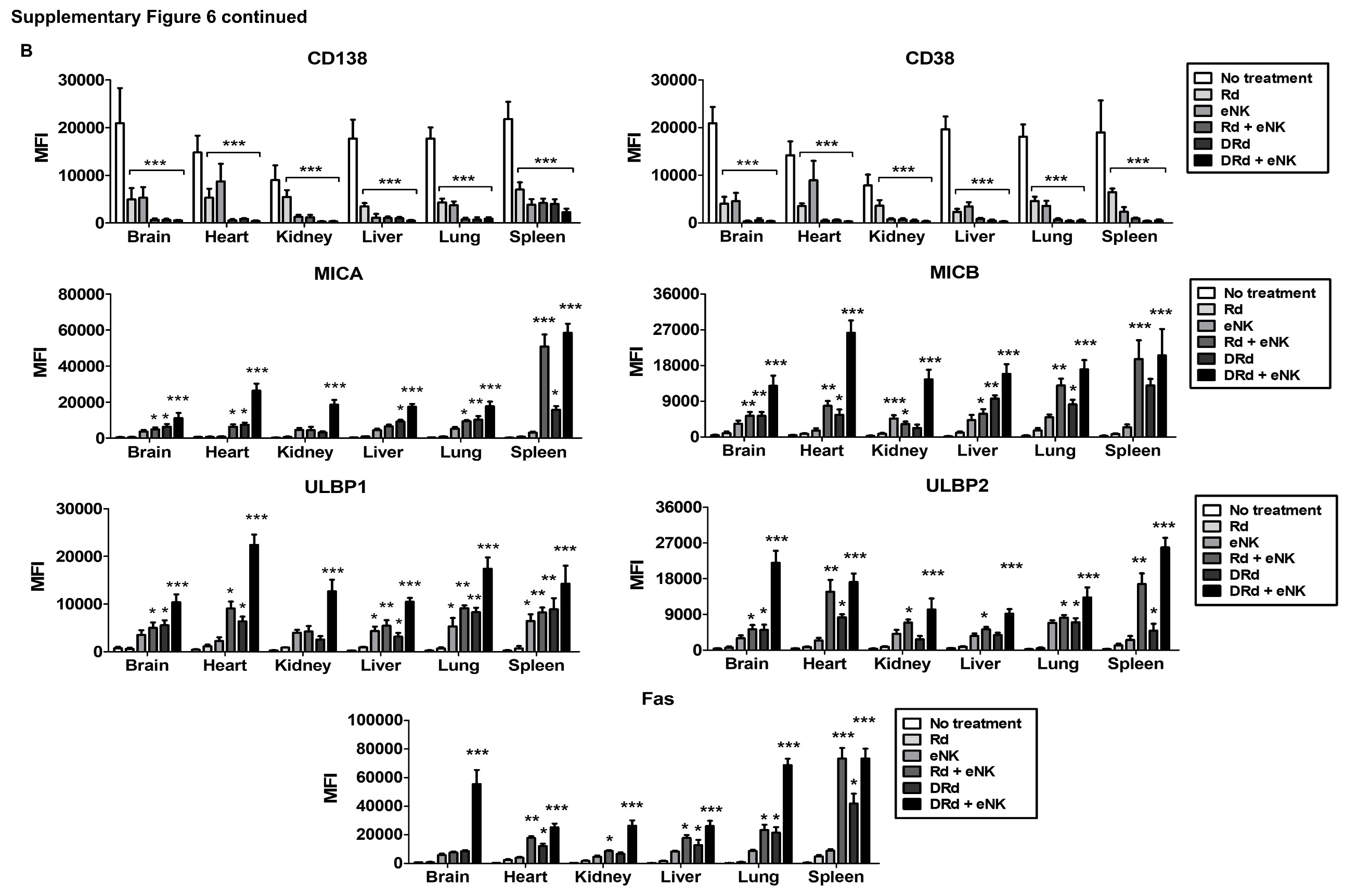


**Supplementary Figure 6. DRd combination with eNK enhances MM clearance and NK activating ligands expression in vivo in RPMI8226-RFP-FLuc xenograft model.** (A) Residual RPMI8226-RFP-FLuc cells and in vivo NK activating ligands expression in the brain, heart, kidney, liver, lung and spleen were evaluated by flow cytometry. Representative histograms showing the expression (MFI value) of CD138, CD38, MICA, MICB, ULBP1, ULBP2, and Fas in brain, heart, kidney, liver, lung, and spleen (B) Quantification (mean ± SD) of residual myeloma cells in the brain, heart, kidney, liver, lung, and spleen. Mice treated with Rd + eNK and DRd + eNK treatment reduced the expression of CD138 and CD38 in all tissues and significantly increased the expression NKG2D activating ligands, such as MICA, MICB, ULBP1, ULBP2, and Fas receptor in brain, heart, kidney, liver, lung, and spleen when compared with the other groups. *p < 0.01, **p < 0.001, ***p < 0.0001.

**Supplement table 2. The list of antibodies used in this study.**

| FITC MOUSE anti human CD3+ | BD | 555332 |
| --- | --- | --- |
| APC MOUSE anti human CD56 clone B159 | BD | 555518 |
| PE Mouse anti human CD16 clone B73⦁1 | BD | 555407 |
| PE Mouse anti human CD69 | BD | 555531 |
| PE Mouse anti human CD94 | BD | 555889 |
| PE Mouse anti human CD337 (NKP30) | BD | 558407 |
| PE Mouse anti human CD336(NKP44) | BD | 558563 |
| PE Mouse anti human CD335(NKP46) | BD | 557991 |
| PE Mouse anti human CD314(NKG2D) | BD | 557940 |
| PE Mouse anti human CD158a | BD | 556063 |
| PE Mouse anti human CD158b | BD | 559785 |
| PE Mouse anti human CD 107a | BD | 555801 |
| PE Mouse anti human perforin | BD | 556437 |
| PE Mouse Anti-Human Granzyme B | BD | 561142 |
| IFN-r Monoclonal antibody(4S⦁B3) | ebioscience | 12-7319-82 |
| FITC mouse IgG1 ,k Isotype control | BD | 555748 |
| APC mouse IgG1 ,k | BD | 555751 |
| PE mouse IgG1 ,k Isotype control | BD | 555749 |
| PE mouse IgG1 ,k Isotype control | BD | 559320 |
| PE mouse IgG1 ,k Isotype control | BD | 554680 |
| PE mouse IgM ,k Isotype control | BD | 555584 |
| PE mouse IgG2b ,kappa Isotype control | BD | 555743 |
| Mouse IgG1 kappa Isotype control, PE | ebioscience | 12-4714-81 |
| Human MICA PE-conjugated Antibody | R&D systems | FAB1300P-100 |
| Human MICB PE-conjugated Antibody | R&D systems | FAB1599P-100 |
| Human ULBP1 PE-conjugated Antibody | R&D systems | FAB1380P |
| Human ULBP2 PE-conjugated Antibody | R&D systems | MAB1298-100 |
| PE anti-human CD253 (TRAIL) Antibody | BioLegend | 308206 |
| PE anti-human CD178 (FASL) Antibody | BioLegend | 306407 |
